# Effect of Low-Frequency, Low-Energy Pulsed Electromagnetic Fields in Neuronal and Microglial Cells Injured with Amyloid-Beta

**DOI:** 10.3390/ijms252312847

**Published:** 2024-11-29

**Authors:** Stefania Merighi, Manuela Nigro, Alessia Travagli, Mercedes Fernandez, Fabrizio Vincenzi, Katia Varani, Silvia Pasquini, Pier Andrea Borea, Simona Salati, Ruggero Cadossi, Stefania Gessi

**Affiliations:** 1 Department of Translational Medicine, University of Ferrara, 44121 Ferrara, Italy; manuela.nigro@unife.it (M.N.); alessia.travagli@unife.it (A.T.); mercedes.fernandez@unife.it (M.F.); fabrizio.vincenzi@unife.it (F.V.); katia.varani@unife.it (K.V.); 2 Department of Chemical, Pharmaceutical and Agricultural Science, University of Ferrara, 44121 Ferrara, Italy; silvia.pasquini@unife.it; 3 University of Ferrara, 44121 Ferrara, Italy; bpa@unife.it; 4 Igea Clinical Biophysics, Medical Division, 41012 Carpi, Italy; s.salati@igeamedical.com (S.S.); r.cadossi@igeamedical.com (R.C.)

**Keywords:** Alzheimer’s disease, low-frequency, low-energy pulsed electromagnetic fields, Aβ peptide, oxidative stress, cell death, microglia, neurons

## Abstract

Alzheimer’s disease (AD) is a neurodegenerative pathology covering about 70% of all cases of dementia. It is associated with neuroinflammation and neuronal cell death, which are involved in disease progression. There is a lack of effective therapies, and halting this process represents a therapeutic challenge. Data in the literature suggest several neuroprotective effects of low-frequency, low-energy pulsed electromagnetic fields (PEMFs) on biological systems, and clinical studies report that PEMF stimulation is safe and well tolerated. The aim of this work is to investigate the effects of PEMF exposure on oxidative stress and cell death in in vitro-injured cellular models of neurons and microglia. SH-SY5Y cells were stimulated by hydrogen peroxide (H_2_O_2_) or amyloid-β (Aβ) peptide, and N9 microglial cells were activated with lipopolysaccharide (LPS) or Aβ peptide. Reactive oxygen production, mitochondrial integrity, and cell death modulation were investigated through 2′,7′-dichlorodihydrofluorescein diacetate (H_2_DCFDA) and 5,5′,6,6′-tetrachloro-1,1′,3,3′-tetraethylbenzimidazolocarbo-cyanine iodide (JC-1) biochemical assays, fluorescence, and MTS experiments. Cells were exposed to PEMFs producing a pulsed signal with the following parameters: pulse duration of 1.3 ms and frequency of 75 Hz. The outcomes demonstrated that PEMFs defended SH-SY5Y cells against Aβ peptide- or H_2_O_2_-induced oxidative stress, mitochondrial damage, and cell death. Furthermore, in microglia activated by LPS or Aβ peptide, they reverted the reduction in mitochondrial potential, oxidative damage, and cell death. Overall, these findings imply that PEMFs influence the redox state of the cells by significantly boosting antioxidant levels in both injured microglia and neuronal in vitro cells mimicking in vitro AD.

## 1. Introduction

Globally, neurological disorders are the second leading cause of mortality and the primary cause of disability. More than 70% of instances of *dementia* are caused by Alzheimer’s disease (AD), which frequently coexists with other neurodegenerative conditions [1]. This ailment is characterized by a steady decline in memory, language, executive function, spatial vision, personality, and behavior, among other cognitive abilities [2]. Within particular regions of the central nervous system, AD is characterized by neuronal death and degeneration, as well as neuroinflammation [3,4].

Given that life expectancy is steadily rising, old age is the biggest risk factor for AD, and as a result, the number of AD patients is also rising, with the number expected to double by 2050 [5]. The most significant neuropathological signs of AD are tau-containing intracellular neurofibrillary tangles and extracellular plaques containing amyloid-β (Aβ) which cause neuronal and synaptic toxicity in the brain [6,7,8]. The resultant neuronal damage may cause memory and cognitive dysfunction. Although the processes behind Aβ-induced neurotoxicity are yet unknown, it has been proposed that many pathways, including oxidative stress, microglial activation, and apoptosis, may be involved. When there is an imbalance between the generation of reactive oxygen species (ROS) and their cellular antioxidant compensation mechanisms, oxidative stress is caused [9]. ROS are molecules that contain extremely reactive oxygen because they have extra electrons damaging other cellular building blocks, such as specific amino acids, proteins, DNA, or lipids. In numerous brain areas, including the hippocampus, which is crucial for memory formation, ROS are known to have a role in the pathophysiology of neurodegenerative illnesses by producing an extracellular deposit of Aβ peptides and intracellular phosphorylated tau fibrils [10,11]. Additionally, it has been established that tau phosphorylation and accumulation are promoted by mitochondrial dysfunction with high ROS levels. As effective regulators of energy metabolism and cell death, mitochondria are essential for neuronal cell survival. Previous research has demonstrated that almost all brain diseases, including AD, are correlated with mitochondrial dysfunction. Alterations in mitochondrial morphology and enzymes have been seen in AD patients [12]. Furthermore, Aβ accumulates and inhibits mitochondrial physiological function, alters the mitochondrial respiratory chain complex, increases ROS accumulation, and lowers adenosine 5′-triphosphate (ATP) generation and mitochondrial membrane potential (MMP), resulting in mitochondrial dysfunction [13]. The accumulation of damaged mitochondria in neurons is a hallmark of AD [14]. Massive neuronal cell death causes shrinkage throughout the cortex and hippocampus in advanced AD, but the reasons are not well elucidated. There are currently no effective medications available that can stop the progression of AD or slow its onset [15]. The N-methyl-D-aspartate (NMDA) receptor antagonist memantine and the cholinesterase inhibitors donepezil, galantamine, and rivastigmine are the only symptomatic drugs actually available; while monoclonal antibodies that target Aβ are under clinical investigation, one of these, Donanemab, received Food and Drug Administration (FDA) approval for the treatment of AD in July 2024 [16,17,18,19]. The capacity to postpone the age at which AD manifests itself by preventative or therapeutic treatments would have considerable benefits, but no medications have been effective in accomplishing this crucial aim. As a result, reducing neuronal damage remains a significant hurdle for contemporary AD treatment.

Pulsed electromagnetic fields (PEMFs) are a non-invasive therapeutic approach for treating a variety of inflammatory illnesses [20]. Interestingly, PEMFs have potent neuroprotective effects on the neurological system [21,22,23,24,25,26]. More specifically, they enhance pro-survival molecules and lower pro-apoptotic mediators, as well as improve angiogenesis [27] and decrease infarct size and neuroinflammation [28]. The signaling that PEMFs trigger to provide neuroprotection against cerebral ischemia has been clarified [22]. Interestingly, in healthy subjects, it was demonstrated that PEMFs can modulate neuronal activity by increasing cortical excitability [21]. An open-label, one-arm, dose-escalation, exploratory study demonstrated the safety and tolerability of PEMFs in patients affected by acute ischemic stroke [29]. Therefore, a multicentric, prospective, randomized, placebo-controlled, double-blind study to evaluate the effectiveness of low-frequency PEMFs in acute ischemic stroke, as a non-invasive and safe tool to promote recovery, was initiated [30]. In addition, PEMFs regulated the expression of microRNA involved in brain signaling and synaptic plasticity in ex vivo human peripheral blood mononuclear cells (PBMCs) from AD patients [31], thus reducing β-secretase and counteracting the formation of Aβ, also suggesting a beneficial effect in AD.

In light of these considerations, the purpose of this work is to examine the function of PEMFs on injured SH-SY5Y neuronal and N9 microglial cells, in order to assess their potential protective effect against oxidative stress, mitochondrial damage and neuronal death. As agents to mimic AD, we used H_2_O_2_ based on its cytotoxic effect and as a source of ROS leading to oxidative stress and Aβ_1–42_ peptide since it is known to increase neuroinflammation and to activate apoptotic pathways inducing a loss of cell viability [32,33].

## 2. Results

### 2.1. Study of PEMF Effects on Oxidative Stress in SH-SY5Y Cells

**Figure 1:**
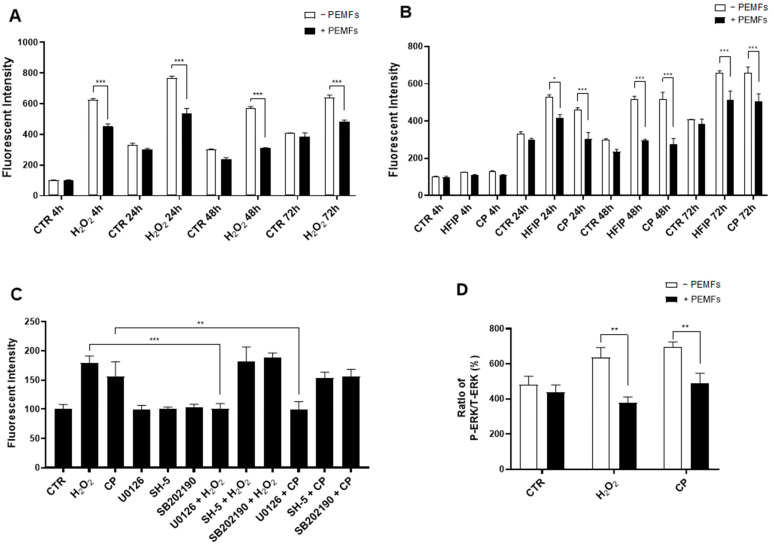
Effect of PEMF exposure on oxidative stress in SH-SY5Y neuronal cells. Effect of PEMFs on ROS levels in SH-SY5Y cells treated with 1 mM H_2_O_2_ or 20 μM HFIP or 20 μM CP (**A**,**B**). Cells were treated with PEMFs continuously for 4, 24, 48, and 72 h. *** *p* < 0.001 and * *p* < 0.05 versus H_2_O_2_, CP, and HFIP without PEMFs (−PEMFs). (**C**) Effect of ERK1/2, p38, and Akt inhibitors at 1 µM (U0126, SB202190, and SH-5, respectively) for 30 min on ROS generation in SH-SY5Y cells treated with 1 mM H_2_O_2_ and 20 μM CP. *** *p* < 0.001 and ** *p* < 0.01 versus H_2_O_2_ and CP. (**D**) Effect of PEMFs on ERK 1/2 activation in neuronal cells treated with 1 mM H_2_O_2_ and 20 μM CP for 20 min. ** *p* < 0.01 versus H_2_O_2_ and CP without PEMFs (−PEMFs). The graphs include mean values ± SEM (n = 3 independent experiments). Statistical analysis was performed using one-way analysis of variance (ANOVA) and Sidak’s multiple comparison test.

ROS play a critical role in neurodegenerative diseases. Oxidative stress appears at the early stage of AD, and Aβ present in the AD brain is known to induce oxidative stress. ROS are able to damage macromolecules including lipids, proteins, and nucleic acids, thereby inducing neuronal death. ROS production was evaluated by measuring intracellular ROS levels with the 2′,7′-dichlorodihydrofluorescein diacetate (H_2_DCFDA) assay. Exposure of SH-SY5Y cells to 1 mM hydrogen peroxide (H_2_O_2_) for 4–72 h induced a significant increase in ROS production as compared to control cells with maximal stimulation after 24 h (Figure 1A). Interestingly, PEMF treatment for 4 to 72 h significantly reduced oxidative stress induced by 1 mM H_2_O_2_ (Figure 1A). To address the effect of Aβ peptide in SH-SY5Y cells, we tested two different amyloid peptides: the toxic fragment (1–42) treated with hexafluoroisopropanol (HFIP) and a chemically modified Aβ (1–42) precursor, O-acyl isopeptide (CP) peptide, with enhanced stability [34], both at 20 µM. With both these products, used in their oligomeric form, we observed a significant effect on ROS production starting at 24 h and continuing until 72 h in SH-SY5Y cells. Importantly, this increase was lowered by continuous PEMF treatment starting from 24 h (Figure 1B). On the basis of these data, we decided to use CP peptide for subsequent experiments. In order to investigate the signaling pathway linked to ROS generation induced by 1 mM H_2_O_2_ and 20 µM CP peptide in SH-SY5Y cells, we evaluated the effect of 1 µM U0126, SB202190, and SH-5, inhibitors of extracellular-signal-regulated kinases (ERKs) 1/2, p38 and protein kinase B (Akt), respectively. Interestingly, our results show that U0126 was able to revert the effect induced by 1 mM H_2_O_2_ and 20 µM CP peptide, whilst SB202190 and SH-5 did not modulate this response (Figure 1C). To evaluate the effect of PEMFs on ERK1/2, cells were incubated with 1 mM H_2_O_2_ and 20 µM CP for 20 min. Interestingly, both dangerous stimuli increased ERK phosphorylation, whilst PEMFs contrasted it (Figure 1D).

### 2.2. Investigation of PEMF Effects on MMP in SH-SY5Y Cells

**Figure 2:**
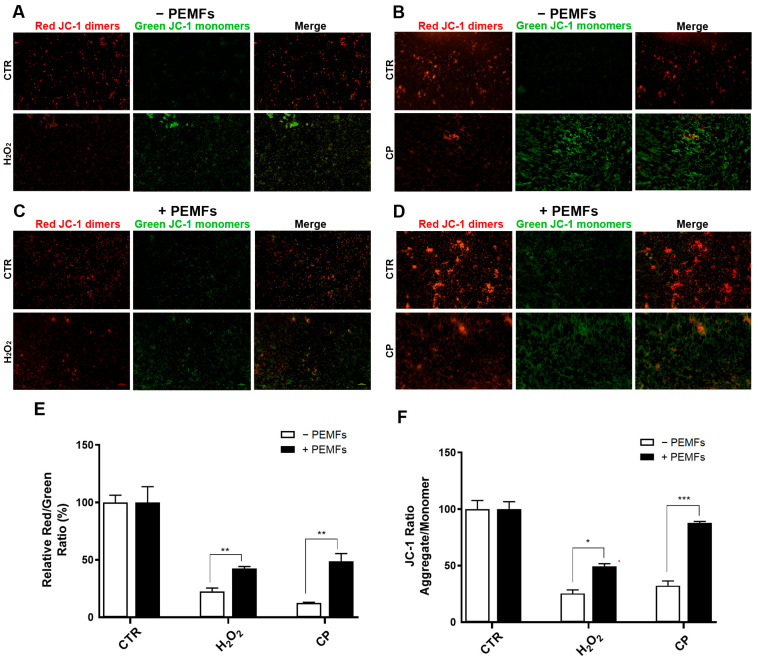
Effect of PEMF exposure on MMP in SH-SY5Y neuronal cells. Cells were treated with 20 μM JC-1 in the absence and presence of PEMFs continuously for 90 min. (**A**) After JC-1 staining, red JC-1 dimers, which represent normal MMP, and green JC-1 monomers, which represent depolarized MMP, can be observed. JC-1 staining of SH-SY5Y cells showed the depolarization of MMP induced by the treatment with 1 mM H_2_O_2_ for 90 min, as can be observed by the huge formation of green monomers. (**C**) The exposition of cells to PEMFs in part reverted this effect since most of the cells showed red dimers which colocalized with the low amount of green dimers formed under this condition. (**B**) The treatment with 20 μM CP for 90 min also induced the depolarization of MMP, as observed by the high number of cells showing green JC-1 monomers. (**D**) SH-SY5Y cell exposure to PEMFs induced a decrease in the amount of green monomers formed. Representative images have been included in panels. Scale bar 100 μM. (**E**) The mean intensity of red and green fluorescent signals was measured from all the captured images using NIS Elements v 5.11 software, and the red/green fluorescence *ratio* was calculated. The relative *ratio* (related to control cells) has been included in the graph where mean values ± SEM (n = 4) are shown. ** *p* < 0.01 versus the H_2_O_2_- and CP-non-exposed groups of cells (−PEMFs). (**F**) Effect of PEMFs on MMP depolarization measured with the JC-1 MMP assay kit, expressed as the JC1 aggregate/monomer ratio, induced by 1 mM H_2_O_2_ and 20 μM CP. * *p* < 0.05 and *** *p* < 0.001 versus H_2_O_2_ and CP without PEMFs (−PEMFs), respectively. The graph includes mean values ± SEM (n = 3). Statistical analysis: one-way analysis of variance (ANOVA) and Sidak’s multiple comparison test.

There is strong evidence that mitochondria contribute to neurodegenerative diseases by producing an excess amount of cytosolic ROS [35]. MMP levels are generally steady in healthy cells; therefore, their reduction indicates a lack of mitochondrial membrane integrity. Such mitochondrial dysfunctions frequently start apoptosis signaling. The MMP was further examined in order to determine whether the neuroprotective effect of PEMFs was linked to mitochondrial activities. The JC-1 MMP of cells was studied by observing cells through a fluorescent microscope. Treating cells with 1 mM H_2_O_2_ and 20 μM CP for 90 min induced mitochondrial depolarization observed by the huge formation of green JC-1 monomers (Figure 2A,B, respectively). Compared with non-PEMF-exposed cells, the PEMF-exposed group of cells showed lower MMP depolarization in the presence of 1 mM H_2_O_2_ and 20 μM CP evidenced by the lower amount of green JC-1 monomers formed (Figure 2C,D, respectively). Figure 2A–D contain representative images of cells from each experimental group. The mean fluorescent intensity of images from all experimental groups was analyzed with NIS Elements software, and the results are presented in Figure 2E.

The mitochondrial health was also evaluated by employing the JC-1 MMP assay kit to detect the role of PEMFs in MMP changes induced by H_2_O_2_ and CP in SH-SY5Y cells. According to the results obtained from the fluorescent microscope JC-1 approach, results show that the MMP was significantly decreased in SH-SY5Y cells, following treatment with 1 mM H_2_O_2_ and 20 μM CP for 90 min, as calculated from the decrease in the *ratio* between fluorescence at 590/530 nm (Figure 2F). Interestingly, PEMFs applied for 90 min were able to revert both H_2_O_2_ and CP effects by increasing the 590/530 nm *ratio* (Figure 2F).

### 2.3. Evaluation of PEMFs’ Effects on Cell Death in Human SH-SY5Y Cells

**Figure 3:**
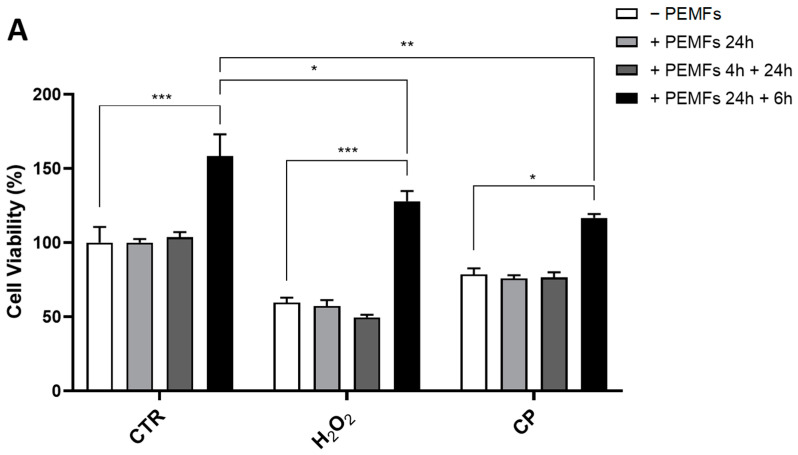

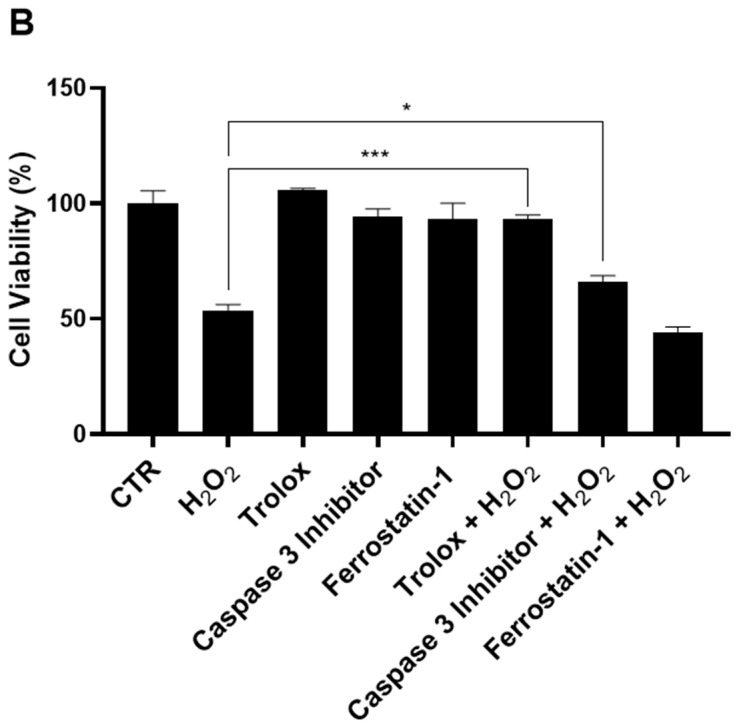
Effect of PEMF exposure on SH-SY5Y neuronal cell viability. (**A**) Effect of different PEMF stimulation timings on the viability of SH-SY5Y cells, treated with 500 μM H_2_O_2_ and 20 μM CP. Cells were exposed to PEMFs as follows: 24 h PEMFs continuously with H_2_O_2_ and CP; pretreated with PEMFs for 4 h, and then continuously with PEMFs, H_2_O_2_ and CP for 24 h; 24 h PEMFs continuously with H_2_O_2_ and CP and 6 h after the termination of PEMF exposure. *** *p* < 0.001 and * *p* < 0.05 versus CTR, H_2_O_2_, and CP without PEMFs (−PEMFs); ** *p* < 0.01 and * *p* < 0.05 versus CTR with PEMFs (+ PEMFs 24 h + 6 h). (**B**) Effect of 500 μM Trolox, 100 μM caspase 3 inhibitors, and 10 μM Fer-1 for 30 min on cell viability of SH-SY5Y cells treated with 500 μM H_2_O_2_. *** *p* < 0.001 and * *p* < 0.05 versus H_2_O_2_. The graphs include mean values ± SEM (n = 3). Statistical analysis was performed.

We assessed how PEMFs affected the cell viability induced by H_2_O_2_ and CP. Our results show that 24 h of treatment with 500 µM H_2_O_2_ and 20 µM CP produced a reduction in cell viability. In detail, H_2_O_2_ and CP induced 40 ± 5% and 16 ± 4% of cell death, respectively (Figure 3A). No protective effects were obtained with PEMF treatment for 24 h or with a pretreatment of PEMFs for 4 h followed by 24 h of continuous stimulation.

Interestingly, in this cell model, 24 h of PEMF treatment showed a pro-survival effect in basal conditions, inducing an increase of 57 ± 7% in cell viability, when the test was performed 6 h post-24 h PEMF treatment. Furthermore, this protective effect was also exerted versus H_2_O_2_ and CP treatments (Figure 3A). In order to investigate whether cell death was a consequence of apoptosis, cells were pretreated with caspase 3 inhibitors for 30 min before the addition of 500 µM H_2_O_2_. In our conditions, the 100 μM caspase 3 inhibitor was able to induce a slight but significant reduction in cell death induced by H_2_O_2_ (46 ± 2% in the absence and 34 ± 1% in the presence of the inhibitor). Therefore, another type of cell death called ferroptosis was addressed by incubating cells with ferrostatin-1 (Fer-1), a specific inhibitor of ferroptosis. In these conditions, 10 µM Fer-1 did not revert H_2_O_2_-induced cell death, suggesting that ferroptosis was not involved (Figure 3B). Furthermore, hypothesizing that cell death could be due to oxidative stress, the ROS scavenger 500 µM Trolox was used. The results indicate that Trolox was able to reduce cell death induced by H_2_O_2_ (46 ± 2% in the absence and 7 ± 2% in the presence of the inhibitor) (Figure 3B).

**Figure 4:**
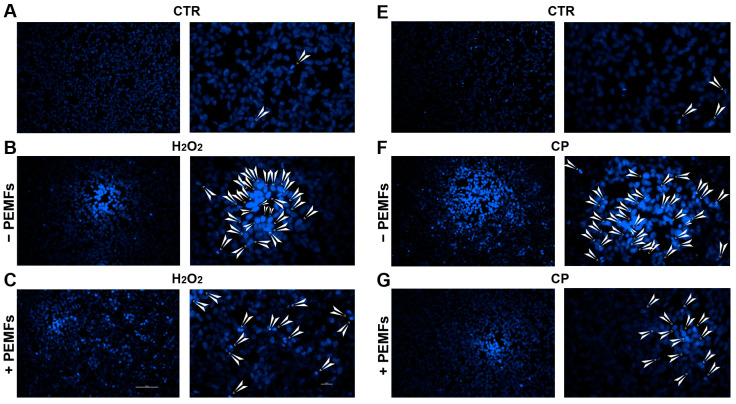

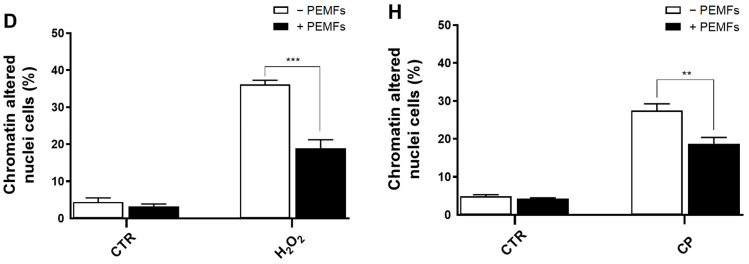
Effect of PEMF exposure on SH-SY5Y neuronal cell viability. Nuclear changes in SH-SY5Y cells after 1 mM H_2_O_2_ or 20 μM CP were studied through the analysis of Hoechst 33342 nuclear staining. The treatment of cells with H_2_O_2_ induced an increase in the number of altered chromatin nuclei cells (**B**) compared with non-treated cells (**A**). The exposure to PEMFs during the treatment significantly protected cells from death (**C**). Representative images of altered chromatin nuclei cells evidenced with white arrowheads are presented in the figure. Scale bars: 100 μM (**left**) and 50 μM (**right**). (**D**) The number of cells with condensed chromatin nuclei in each experimental condition was analyzed, and it is expressed as the percentage of the total number of cells in the graph where mean values ± SEM (n = 3) are included. *** *p* < 0.001 versus H_2_O_2_ without PEMFs (−PEMFs). The treatment with CP also induced an increase in the number of altered chromatin nuclei cells (**F**) compared with control cells (**E**), which was significantly lower in the group of cells exposed to PEMFs (**G**). The graph (**H**) includes mean values ± SEM (n = 3) of altered chromatin nuclei cell percentage. ** *p* < 0.01 versus CP without PEMFs (−PEMFs). Statistical analysis: one-way analysis of variance (ANOVA) and Sidak’s multiple comparison test.

The nuclear staining of SH-SY5Y cells with Hoechst 33342 evidenced a high number of cells with condensed and/or fragmented chromatin nuclei in response to 1 mM H_2_O_2_ for 90 min, compared with control cells which showed very few altered nuclei (Figure 4A,B). When cells were exposed to PEMFs, the number of nuclei-altered cells decreased (Figure 4C). The results of altered nuclei quantification expressed as a percentage of the total number of nuclei have been included in panel D (Figure 4). Interestingly, a similar pattern of cell death was found by evaluating the effect of 20 µM CP on SH-SY5Y cells, in the absence or presence of PEMFs (Figure 4E–G). Quantification of apoptotic nuclei demonstrates their reduction following PEMF application (Figure 4H).

### 2.4. Study of PEMF Effects on Oxidative Stress in N9 Microglial Cells

**Figure 5:**
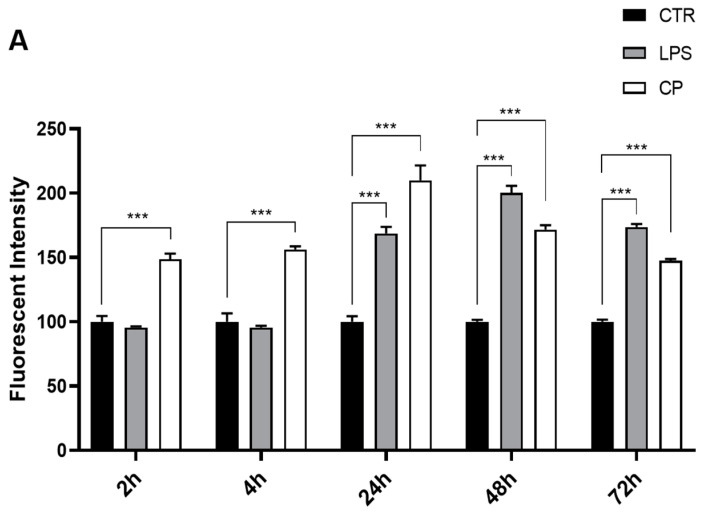

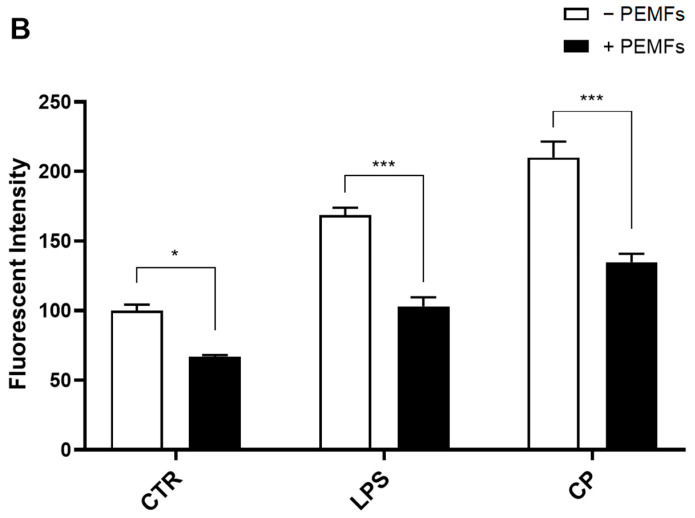
Effect of PEMF exposure on oxidative stress in N9 microglial cells. (**A**) Effect on ROS levels in N9 cells treated with 1 μg/mL LPS and 20 μM CP for 2, 4, 24, 48, and 72 h. *** *p* < 0.001 versus CTR. (**B**) Effect of PEMFs on ROS levels in N9 cells after treatment with 1 μg/mL LPS and 20 μM CP for 24 h. Cells were treated with PEMFs continuously for 24 h. *** *p* < 0.001 and * *p* < 0.05 versus CTR, LPS, and CP without PEMFs (−PEMFs). The graphs include mean values ± SEM (n = 3). Statistical analysis: one-way analysis of variance (ANOVA) and Sidak’s multiple comparison test.

We further investigated the impact of PEMFs on the regulation of ROS levels in microglial cells, as the development of AD is linked to ROS accumulation. ROS production was evaluated by measuring intracellular ROS levels with the H_2_DCFDA assay for 2–72 h (Figure 5A). Exposure of N9 microglial cells to 1 μg/mL LPS and 20 μM CP induced a significant increase in ROS production as compared to control cells, with a maximal effect of CP at 24 h of 218 ± 24% (Figure 5A). Interestingly, 24 h of PEMF treatment significantly decreased basal and LPS- or CP-induced ROS levels in N9 cells by 33, 39, and 36%, respectively, suggesting that PEMFs provided a basal protective effect, independent of the stimulus used to increase ROS (Figure 5B).

### 2.5. Investigation of PEMF Effects on MMP in N9 Microglial Cells

**Figure 6:**
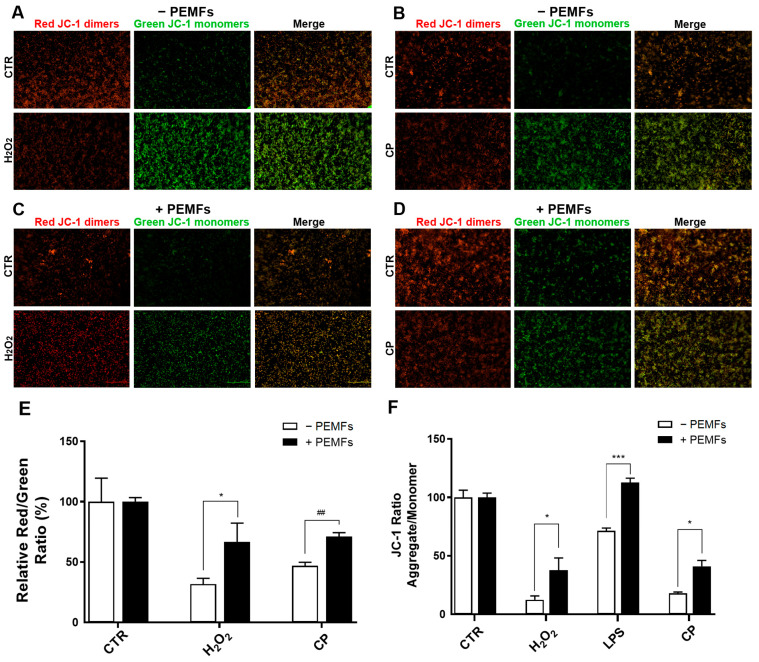
Effect of PEMF exposure on MMP in murine N9 cells. (**A**) JC-1 staining showed red dimers in control cells representing normal MMP. The treatment of N9 cells with 1 mM H_2_O_2_ for 90 min induced a massive formation of green monomers. (**C**) The exposition of cells to PEMFs in part reverted the effect of H_2_O_2_ as observed by the decrease in the amount of green monomers and the increase in the red dimer fluorescence. (**B**) The treatment of N9 cells with 20 μM CP for 90 min induced the depolarization of MMP as observed by the formation of green monomers. (**D**) This effect was attenuated in the group of N9 cells exposed to PEMFs during the treatment. Scale bar 1000 μM. (**E**) The mean intensity of red and green fluorescent signals was measured from all the captured images, and the red/green ratio was calculated. The relative *ratio* (related to control cells) is included in the graph where mean values ± SEM (n = 3) are shown. * *p* < 0.05 versus H_2_O_2_ and ^##^ *p* < 0.01 versus CP without PEMFs (−PEMFs). (**F**) Effect of PEMF exposure on MMP depolarization, measured with the JC-1 MMP assay kit, expressed as JC1 aggregate/monomer *ratio*, induced by 1 mM H_2_O_2_, 1 μg/mL LPS, and 20 μM CP in microglial cells. Cells were treated with JC-1 20 μM in the absence and presence of PEMFs continuously for 90 min. *** *p* < 0.001; * *p* < 0.05 versus H_2_O_2_, LPS and CP without PEMFs (−PEMFs). The graph shows mean values ± SEM (n = 3). Statistical analysis: (*) one-way analysis of variance (ANOVA) and Sidak’s multiple comparison test; (#) Student’s *t* test.

Mitochondrial function was assessed using the JC-1 probe to identify the effects of PEMFs on MMP alterations driven by different injuries. Using a fluorescence microscope, the MMP of N9 cells loaded with JC-1 was investigated. Cells treated with 1 mM H_2_O_2_ and 20 μM CP for 90 min showed signs of mitochondrial depolarization, as evidenced by the massive production of green JC-1 monomers (Figure 6A,B, respectively). The group of cells subjected to PEMF exposition exhibited reduced levels of MMP depolarization, as indicated by the decreased formation of green JC-1 monomers, the increase in red dimers and the colocalization of both dimers and monomers (Figure 6C,D, respectively). Figure 6A–D contains representative images of cells from each experimental group. The mean fluorescent intensity of images from all experimental groups was analyzed with the NIS Elements software, and the results are presented in Figure 6E. The mitochondrial functionality was also evaluated, through a JC1 MMP assay kit, to detect the functions of PEMFs on MMP changes induced by LPS, H_2_O_2_, and CP in N9 cells.

According to the results obtained from the fluorescence microscope JC-1 method, 1 mM H_2_O_2_ and 20 μM CP for 90 min produced MMP damage that was reversed by PEMFs in N9 cells (Figure 6F). In addition, a similar behavior was found following insult with 1 μg/mL LPS treatment.

### 2.6. Evaluation of PEMF Effects on Cell Death in Murine N9 Cells

**Figure 7:**
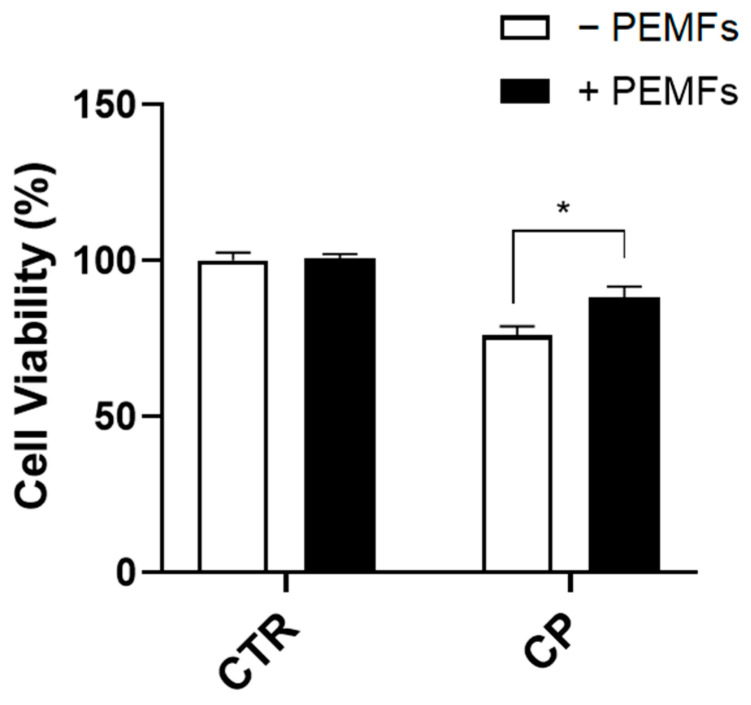
Effect of PEMF exposure on microglia N9 cell viability. N9 cells were treated with 20 μM CP and exposed to PEMFs for 24 h continuously with CP. * *p* < 0.05 versus CP without PEMFs (−PEMFs). The graph includes mean values ± SEM (n = 3). Statistical analysis: one-way analysis of variance (ANOVA) and Sidak’s multiple comparison test.

We investigated the impact of PEMFs on CP-influenced N9 cell viability. Our findings reveal that 24 h of exposure to 20 µM CP resulted in a shift in cell viability from 76 ± 5 to 88 ± 6% in the absence and presence of PEMFs, respectively (Figure 7).

**Figure 8:**
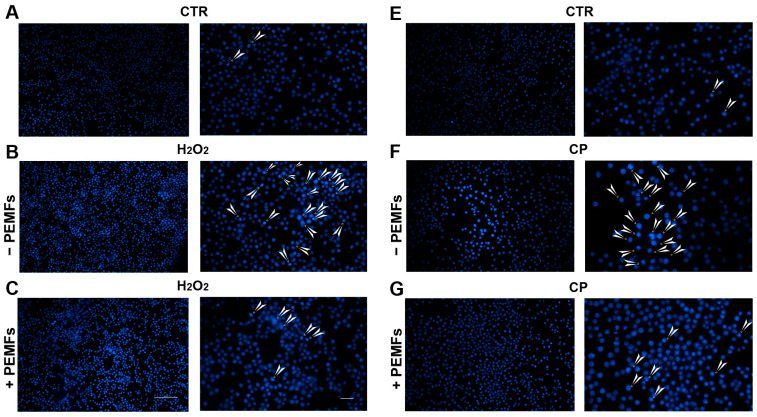

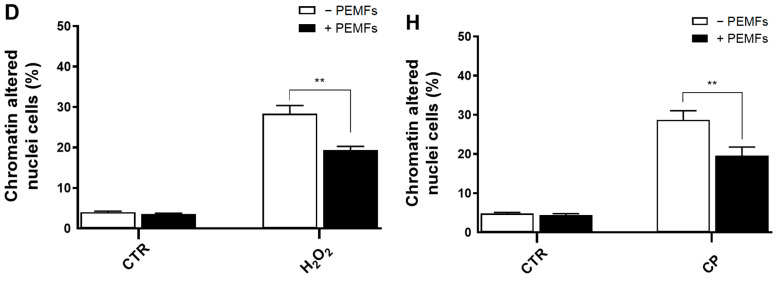
Effect of PEMF exposure on murine N9 cell death. Hoechst 33342 nuclear staining of N9 cells after 1 mM H_2_O_2_ or 20 μM CP was performed to study cell death. The treatment of cells with H_2_O_2_ induced an increase in the number of altered chromatin nuclei cells evidenced by white arrowheads (**B**) compared with non-treated cells (**A**). PEMF exposure of cells during the treatment significantly decreased the number of nuclei-altered cells (**C**). Representative images of altered chromatin nuclei cells are included in the figure. Scale bars: 100 μM (left) and 50 μM (right). (**D**) The percentage of condensed chromatin nuclei referring to the total number of cells from each experimental group (mean values ± SEM, n = 3) is included in the graph. ** *p* < 0.01 versus H_2_O_2_ without PEMFs (−PEMFs). (**F**) The treatment with CP also induced an increase in the number of altered chromatin nuclei cells compared with control cells (**E**), which was significantly lower in the group of cells exposed to PEMFs (**G**). (**H**) The graph includes mean values ± SEM (n = 4) of altered chromatin nuclei cell percentage. ** *p* < 0.01 versus H_2_O_2_ and CP without PEMFs (−PEMFs). Statistical analysis was performed using one-way analysis of variance (ANOVA) and Sidak’s multiple comparison test.

The treatment with 1 mM H_2_O_2_ for 90 min increased the number of altered chromatin nuclei cells compared with control cells (Figure 8A,B). PEMF exposure of cells showed a cytoprotective effect attenuating H_2_O_2_-induced altered nuclei in N9 cells (Figure 8B). The number of altered nuclei was quantified, and results, expressed as a percentage of the total number of nuclei, are included in panel D (Figure 8D). Interestingly, when 20 µM CP was evaluated in N9 cells with or without PEMFs, a comparable pattern of H_2_O_2_-induced cell death was discovered, as Figure 8 illustrates. Apoptotic nuclei are quantified and show a decrease after PEMF administration (Figure 8H).

## 3. Discussion

Neurodegenerative diseases like AD have been linked to neuronal loss due to oxidative stress [36]. High levels of ROS resulting from an imbalance between their generation and the antioxidant defense system are referred to as oxidative stress. This event is observed in a variety of physiological and biological reactions, including antibacterial and antiviral effects, cell growth, proliferation, differentiation, survival, aging, and death. Excessive production of ROS can result in genetic alterations that affect genes crucial for the antioxidant defense system and DNA repair mechanisms. These same genes can cause mutations in mitochondrial DNA, deterioration of mitochondrial membrane permeability and respiratory function, and disturbance of Ca^2+^ homeostasis. Thus, several major pathological processes in AD, including Aβ-induced neurotoxicity, tau-induced neurofibrillary tangles, and mitochondrial dysfunction are associated with oxidative stress involving ROS and reactive nitrogen species [37]. The brain needs to keep an equilibrium between antioxidants and oxidative stressors. All metabolic activities result in the production of ROS, which are essential to cells’ healthy function. However, excessive ROS results in lipid peroxidation and nerve apoptosis in addition to destroying the DNA strands.

H_2_O_2_ is a highly reactive ROS and one of the most significant oxidative stress mediators that has been shown to cause neuronal damage through the induction of mitochondria-related apoptotic signals. To replicate in vitro oxidative stress in a variety of cell types, H_2_O_2_ has been frequently used as a neurotoxic challenge paradigm. Another important molecule in the neurodegenerative cascades of AD has been identified as Aβ. Since Aβ directly causes neuronal death, it is likely that Aβ-induced neurotoxicity plays a significant part in AD neurodegeneration. Specifically, in the AD brain, free radicals are created when β-amyloid is deposited, which first causes inflammation and damage before resulting in the death of nerve cells. It has been demonstrated that there are several ways in which Aβ causes neuronal cell death, including necrosis, apoptosis, and necroptosis [38].

In this work, exogenous H_2_O_2_ and Aβ_1–42_ amyloid peptide were used to damage human SH-SY5Y neuronal cells in order to mimic the environment of the AD brain. The literature shows many articles describing studies using in vitro injuries to mimic AD pathology. Different substances were used for the in vitro development of AD, targeting mechanisms of neurodegeneration [39]. Some environmental agents used were aluminum chloride [40], particulate matter [41,42], H_2_O_2_ [43,44], etc., while others used were chemical agents, such as Aβ peptides [32,33,45,46]. Although none of these injuries can accurately reproduce all the characteristics of human AD pathology, these in vitro strategies are good tools to study cellular events associated with this disease and to gain deeper insights into the disease’s pathophysiology [39]. Moreover, each agent used to mimic AD in vitro might act through different signaling pathways, thus replicating particular stages of AD alterations and associated molecular mechanisms [39].

Finding novel and effective strategies to reduce the neurotoxicity caused by oxidative stress may prove beneficial in both preventing and treating AD. Few studies have explored the connection between exposure to PEMFs and the onset of AD in this scenario. Early studies reported that PEMF stimulation significantly reduced hypoxia-induced cell death in astrocytes as well as in neuronal SH-SY5Y and PC12 cells [22,24]. Moreover, in microglial cells, PEMF exposure mediated a significant reduction in ROS and cytokine production [25].

Therefore, the possible neuroprotective benefits of PEMFs have been examined in SH-SY5Y and microglia cells. According to the literature data, the findings of this research indicate that stimulation of SH-SY5Y cells with both H_2_O_2_ and CP led to an increase in cytosolic ROS. Interestingly, our results indicate that PEMF treatment for 24 h significantly alleviates H_2_O_2_- and CP-induced ROS increase.

Mitogen-activated protein kinase (MAPK) and PI3K/Akt pathways are important signaling pathways involved in various cellular activities, including responses to oxidative stress. The evidence that ROS can activate MAPK pathways at the cellular level is based on a variety of research. For example, biological stimuli capable of creating ROS such as H_2_O_2_ have been shown to activate MAPK pathways in many kinds of cell types. Accordingly, antioxidants and inhibitors of ROS-producing enzyme systems prevent MAPK activation. The possible mechanisms by which ROS can activate MAPK pathways, based on their oxidation potentials, include oxidative changes in MAPK signaling proteins or inactivation of mitogen-activated protein kinase phosphatase that prevents activation of the route [47,48].

In neurons and microglia, the impact of PEMFs on MAPK and Akt signaling has not been thoroughly studied. It has been reported that in NGF-stimulated hypoxic PC12 cells, for instance, PEMFs were seen to activate the p38 MAPK pathway in order to support neuronal survival [22]. However, for the activation of adult neurogenesis, PEMF elevated ERK phosphorylation in human bone marrow mesenchymal stem cells [49]. Indeed, activation of the Akt and BDNF signaling pathways was responsible for PEMF protection against ischemic stroke in previous studies [50]. Accordingly, a metabolomic investigation in SH-SY5Y, a Parkinson’s disease model, found that PEMFs changed the metabolome profile and boosted the PI3K/AKT pathway, implying a function for them in autophagy inhibition and dopamine neuron survival [51]. In addition, PEMFs inhibited proinflammatory cytokine expression in N9 microglial cells by upregulating JNK phosphorylation [25].

Therefore, the regulating effect of PEMFs on MAPK and Akt in ROS production was evaluated in SH-SY5Y cells. Interestingly, our data show that ROS generation caused by H_2_O_2_ was reversed by an ERK inhibitor but not by p38 and Akt blockers. In line with the literature [52,53,54], we observed that CP and H_2_O_2_ both activate ERK, and for the first time, we discovered that PEMFs were able to reduce this phosphorylation. This suggests that PEMFs may be used to induce ERK inactivation, offering some neuroprotection. The exact mechanism by which PEMFs reduce H_2_O_2_-induced ERK phosphorylation is not fully understood and is a topic of ongoing research. It is known that H_2_O_2_ is linked to redox signaling, including the reversible oxidation of redox-sensitive cysteine residues in enzymes and transcription factors, which alters their activity and may be contrasted by PEMFs [36]. In addition, PEMFs dramatically reduce inflammatory cytokines such as tumor necrosis factor α (TNF-α), interleukin-1β (IL-1β), interleukin-6 (IL-6), and interleukin-8 (IL-8) [25]. These cytokines are known to affect ERK activity; therefore, lowering them may possibly lead to a decline in ERK, which has been documented to play a harmful role in AD [55,56]. Activation of MAPKs leads to the transfer of inflammatory signals into the nucleus, including the nuclear factor kappa B (NF-κB) pathway, inducible nitric oxide synthase (iNOS), cyclooxygenase-2 (COX-2) production, and inflammatory cytokines, which contribute to neurodegeneration. In particular, proinflammatory cytokines, such as TNF-α, can boost ROS generation through the NADPH oxidase route, activating NF-κB in glial cells leading to a prolonged proinflammatory response and damaging mitochondria in neurons.

Therefore, our data suggest that MMP, which is important for cell health, was diminished by H_2_O_2_ or CP treatments, thereby affecting neuronal viability. Both these detrimental agents were contrasted by PEMFs. This is of interest because broken mitochondria may accelerate the degenerative process of AD [57]. When the effects of H_2_O_2_ and CP on cell death were investigated, we found that both these stimuli were able to induce a reduction in neuron viability. Importantly, we discovered that PEMFs alone have a pro-survival impact when cell viability was assessed 6 h after administration for 24 h. Thus, 24 h of PEMF exposure reduces H_2_O_2_- and CP-induced ROS levels, but it does not instantly boost cell viability. As a consequence, we propose that this treatment, in addition to partially reducing ROS, may also enhance pro-survival proteins such as BDNF, which take time to synthesize. This would explain why a 6 h gap without PEMFs is required following 24 h of PEMF therapy to exhibit protection.

A detailed look into SH-SY5Y cell death revealed that both apoptosis and ROS generation were involved, but not ferroptosis. This was demonstrated by pretreating the cells with a caspase 3 inhibitor, and using Trolox and Fer-1 as ROS and ferroptosis blockers, respectively. Accordingly, the nuclear staining of SH-SY5Y cells with Hoechst 33342 confirmed apoptosis, showing a high number of cells with condensed and/or fragmented chromatin nuclei. Considering that the exact mechanism of neuronal loss in AD is unknown, this is intriguing. It is clear that a variety of cell death mechanisms may either independently or concurrently contribute to the loss of neurons. The most studied mechanism of cell death in AD is apoptosis, which was the first type of controlled cell death to be identified [58]. Apoptosis is a type of non-inflammatory cell death that results in the removal of the cell without any intracellular components being released. It is carried out by two main mechanisms—intrinsic and extrinsic apoptosis—and it is elicited by a variety of triggers. These routes come together when caspase 3 is cleaved. Cleaved caspase 3 engages proteins that cause nuclear condensation, DNA fragmentation, cytoplasmic condensation, and membrane blebbing. However, post-mortem tissue samples from AD patients do not show extensive neuronal apoptosis [58]. Therefore, other forms of cell death that have recently been discovered may potentially have an impact on AD neuronal degeneration, and our results point towards a crucial role of a dangerous ROS-mediated effect. Increased focus on the process of AD neuron death will contribute to our knowledge of the pathophysiology of this neurodegenerative disease and might result in the development of novel treatment approaches. In this context, PEMFs have emerged as a crucial tool for limiting cell death.

Interestingly, the effect of PEMFs on oxidative damage was not limited to neuronal cells but was also observed in microglia. Indeed, our results show that, following stimulation of microglia with LPS and CP, there was an increase in mitochondrial damage and ROS, which were reduced after exposure to PEMFs. Indeed, microglia play a dual role in AD. On one hand, they proliferate and become activated in response to amyloid plaques, contributing to inflammation and neurodegeneration. On the other hand, they also play a role in plaque clearance. Therefore, reducing microglial death could potentially enhance their beneficial effects. Importantly PEMFs were able to reduce the death of injured microglial cells. Accordingly, a similar role in promoting microglial survival has been exploited by the development of a humanized monoclonal antibody, AL002, activating a triggering receptor expressed on myeloid cells 2 (TREM2) and inducing microglia proliferation [59].

We previously demonstrated that PEMFs inhibited proinflammatory cytokine release, phagocytosis, and cell invasion in microglial cells [25]. In the current study, we report that they significantly diminished the levels of ROS in both neurons and microglia, improved mitochondrial function, and reduced cell death, providing substantial evidence of their efficacy in mitigating neuronal damage. This is of importance in the neurodegenerative process, considering that M1 microglial cells release proinflammatory mediators and ROS, thus contributing to neuronal death. Therefore, the effects of PEMFs in both neurons and microglia imply that they could affect the overall redox status of the cells. This appears to be a significant result given that the therapeutic impact of most antioxidant molecules is restricted, due to their inability to penetrate the blood–brain barrier. This is evident in the clinical trial failure of antioxidant treatments. As a result, an effective AD treatment is urgently required, and antioxidant therapy may have wide-ranging applications in inflammaging, a persistent condition of inflammation linked to aging.

### Limitations of the Study

Since we only employed in vitro models in our study, it is not possible to extend our results to live organisms or human patients. However, in vitro models must be used to thoroughly define the molecular mechanism of their impacts before planning investigations in animal models to obtain direct information about the complete organism.

## 4. Materials and Methods

### 4.1. Drugs and Materials

Penicillin and streptomycin, L-glutamine, Dulbecco’s Modified Eagle’s Medium/Nutrient Mixture F12 Ham (DMEM F12), H_2_O_2_, LPS (from *Escherichia coli*, serotype 055:B5), phosphate-buffered saline (PBS), acetic acid, and anhydrous dimethyl sulfoxide (DMSO) were purchased from Merck (Milan, Italy). The assay kit for ROS evaluation was purchased from Canvax Biotech (Voden, MB, Italy). CellTiter 96^®^ AQueous One Solution MTS cell proliferation assay was obtained from Promega (Milan, Italy). Human neuronal SH-SY5Y cells were purchased from the American Type Culture Collection (ATCC) (Manassas, VA, USA). Iscove’s Modified Dulbecco’s Medium (IMDM), Minimum Essential Medium Eagle (EMEM), fetal bovine serum (FBS), and Australian FBS were obtained from EuroClone (Milan, Italy). Finally, HFIP Aβ_1–42_ peptide was obtained from Bachem AG (Bubendorf, Switzerland), and CP Aβ_1–42_ was obtained from GenScript Biotech (Twin Helix, Milan, Italy).

### 4.2. Electromagnetic Field Exposure System

Human neuronal SH-SY5Y cells and N9 microglial cells were exposed to PEMFs generated by a pair of rectangular horizontal coils (14 × 23 cm), each made of 1.400 turns of copper wire placed opposite to each other. The complete exposure system has previously been described in detail. The culture was placed between this pair of coils so that the plane of the coils was perpendicular to the culture flasks. The coils were powered by the PEMF generator system (IGEA, Carpi, Italy) used in previous studies [60,61], which produced a pulsed signal with the following parameters: pulse duration of 1.3 ms and frequency of 75 Hz, yielding a 0.1 duty cycle. The peak intensity of the magnetic field and peak intensity of the induced electric voltage were detected in the air between two coils from one side to the other, at the level of the culture flasks. The peak values measured between two coils in the air had a maximum variation of 1% in the whole area in which the culture flasks were placed. The dimensions of the flasks were 9.2 × 8.2 cm with 10 mL of medium. The peak intensity of the magnetic field was 1.5 ± 0.2 mT and it was detected using the Hall probe (HTD61-0608-05-T, F.W. Bell, Sypris Solutions, Louisville, KY, USA) of a gaussmeter (DG500, Laboratorio Elettrofisico, Milan, Italy) with a reading sensitivity of 0.2%. The corresponding peak amplitude of the induced electric voltage was 2.0 ± 0.5 mV. It was detected using a standard coil probe (50 turns, 0.5 cm internal diameter of the coil probe, 0.2 mm copper diameter) and the temporal pattern of the signal was displayed using a digital oscilloscope (Le Croy, Chestnut Ridge, NY, USA). The shape of the induced electric voltage and its impulse length were kept constant.

### 4.3. Cell Cultures

Human neuronal SH-SY5Y cells were cultured in DMEM F12 and EMEM supplemented with 10% FBS, L-glutamine (2 mM), penicillin (100 U/mL), and streptomycin (100 μg/mL); the cultures were maintained at 37 °C in a humidified atmosphere with 5% CO_2_ [62]. The N9 murine microglial cell lines, kindly provided by Prof. Ricciardi-Castagnoli, were cultured in IMDM, supplemented with 10% heat-inactivated Australian FBS, L-glutamine (2 mM), penicillin (100 U/mL), and streptomycin (100 μg/mL) [25]. Cells were grown in a humidified environment containing 5% CO_2_ at a constant temperature of 37 °C.

Before the experiments, the cell culture medium was replaced with a fresh serum-free medium to minimize the interference of growth factors in the serum with signal transduction.

### 4.4. Cellular Treatment

H_2_O_2_ is an oxygen radical causing neuronal toxicity and was used as positive control at 500 and 1000 μM. LPS, a cell wall component of Gram-negative bacteria, is a potent activator of glia. β-amyloid peptide (Aβ) in the range of 1–50 µM was used as an insult to mimic AD pathology through induction of toxicity and inflammation. Cells were treated with H_2_O_2_, LPS, or Aβ and then incubated in the presence or in the absence of PEMFs.

### 4.5. Preparation of HFIP-Treated Aβ Peptide Stocks

Prior to use, the peptide was removed from a −20 °C freezer and allowed to come to RT. A 5 mM Aβ DMSO stock was prepared by adding fresh anhydrous DMSO. To ensure complete resuspension of peptide, the solution was pipetted thoroughly. Then, the solution was vortexed well (~30 s) and pulsed in a microcentrifuge to collect it at the bottom of the tube (peptide was not stored as a DMSO stock for more than 1 h to avoid protofibril formation). Then, 5 mM Aβ DMSO solution was sonicated for 10 min in a bath sonicator. For oligomeric Aβ preparation (5 mM Aβ_1–42_), DMSO solution was diluted with PBS pH 7.4 to a final concentration of 100 μM. Then, the solution was vortexed for 15 s, and aggregated on an orbital shaker (~300 rpm) at room temperature (RT) for 1 h 30 min.

### 4.6. Preparation of O-Acyl Isopeptide (CP) Stocks

The CP peptide of Aβ_1–42_, possessing an ester bond at the Gly(25)-Ser(26) sequence, is a water-soluble and non-aggregative precursor molecule and is capable of producing Aβ_1–42_ monomers. In addition, the O-acyl isopeptide is promptly converted to Aβ_1–42_ (t_1/2_ 10 s) at pH 7.4, via an O-to-N intramolecular acyl migration reaction [63]. Then, 100 μM CP was converted to native peptide by adding PBS pH 7.4 and aggregated on an orbital shaker (~300 rpm) at RT for 1 h 30 min.

### 4.7. ROS Detection Assay (DCFDA/H_2_DCFDA)

The antioxidant potential of PEMFs was tested in SH-SY5Y neuronal and N9 microglial cells by the 2′,7′-dichlorofluorescein diacetate (H_2_DCFDA) assay. In detail, SH-SY5Y (6 × 10^3^ cells/well) and N9 (3 × 10^4^ cells/well) were seeded in a black 96-well plate and incubated overnight. Subsequently, the medium of each well was removed, and 100 µL of 20 µM H_2_DCFDA solution was added. The plate was then incubated in the dark at 37 °C. After 1 h incubation, a wash with Assay Buffer 1× was performed. Subsequently, SH-SY5Y cells were treated with H_2_O_2_, CP, and HFIP in a serum-free medium and were then exposed to PEMFs for 4–72 h. Inhibitors of signaling U0126, SB202190, and SH5 were preincubated for 30 min and then cells were injured with H_2_O_2_ and CP for 24 h. N9 cells were treated with LPS and CP and then exposed to PEMFs for 24 h. The fluorescence was read with the Ensight multimode plate reader at an excitation of 485 nm and an emission of 538 nm (Perkin Elmer, Milan, Italy) at different times.

### 4.8. ELISA Assay

The levels of ERK1/ERK2 (Total/Phospho) proteins were determined by an ERK1/ERK2 (total/phospho) multispecies InstantOne^TM^ ELISA kit. The protein levels were measured according to the manufacturer’s instructions. In brief, SH-SY5Y neuronal cells (3 × 10^5^ cells/well) were plated in 24-well dishes and allowed to attach overnight. Subsequently, cells were treated with H_2_O_2_ and CP, in the presence or absence of PEMFs for 20 min. At the end of the treatment, the cells were lysed using 300 μL of lysis buffer 1×, and 50 μL of cell lysate was added to the InstantOne^TM^ assay microplate. Then, 50 μL of freshly prepared antibody cocktail (containing the capture and detection antibodies) was added into each well and after 1 h of incubation at RT with shaking (~300 rpm), three washes were performed. Finally, 100 μL of detection reagent was added to each well for 30 min and the optical density was detected by the Ensight multimode plate reader (Perkin Elmer, Milan, Italy) at a wavelength of 450 nm.

### 4.9. JC-1 Test

For the measurement of MMP, we used the JC-1—MMP assay kit. This kit contains JC-1, a cationic dye that accumulates in mitochondria. At low concentrations (due to low mitochondrial membrane potential), JC-1 is predominantly a monomer that yields green fluorescence with emission at 530 ± 15 nm. At high concentrations (due to high mitochondrial membrane potential), the dye aggregates yield a red- to orange-colored emission (590 ± 17.5 nm). Therefore, a decrease in the aggregate fluorescent count is indicative of depolarization, whereas an increase is indicative of hyperpolarization.

In detail, SH-SY5Y (2 × 10^4^ cells/well) and N9 (5 × 10^4^ cells/well) were seeded in a black 96-well plate and incubated overnight. Subsequently, the medium of each well was removed and 100 μL of JC-1 solution was added. The plate was then incubated in the dark at 37 °C. After 30 min of incubation, two wash steps with Dilution Buffer 1× were performed. Then, treatments with H_2_O_2_, CP, and LPS for 90 min with or without PEMFs were performed in the second wash step with Dilution Buffer 1×. The fluorescence was read with the Ensight multimode plate reader (Perkin Elmer, Milan, Italy).

MMP was also analyzed using a fluorescence microscope with the same kit. For this type of analysis, the SH-SY5Y and N9 cells (2 × 10^5^ cells/well) were seeded in 8-well culture/chamber slides and treated as described above (Nunc Lab-Tek Chamber Slides, Thermo Fisher Scientific, Italy). Fluorescent images were acquired using a fluorescent microscope (Nikon Eclipse 5i) equipped with a cooled charge-coupled device (CCD) camera (Nikon DS-Qi1). The images were analyzed using the software NIS Elements v 5.11.

### 4.10. MTS Assay

**Scheme 1:**
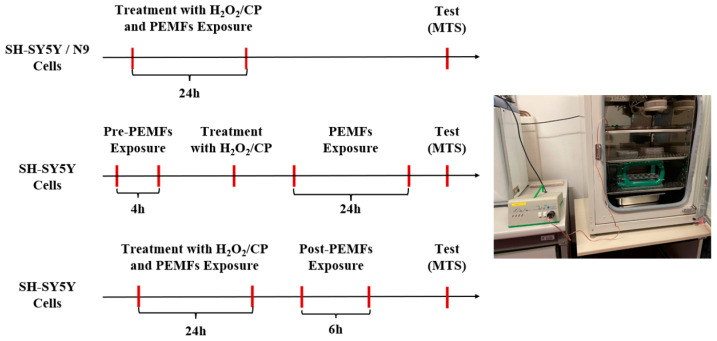
Schematic experimental design for study on SH-SY5Y and N9 cells and PEMF exposure system.

The MTS assay was performed to determine cell viability according to the manufacturer’s protocol from the CellTiter 96^®^ AQueous One Solution cell proliferation assay (Promega Italia Srl, Milan, Italy). In particular, SH-SY5Y and N9 (3 × 10^4^ cells/well) were seeded in a 96-well plate and incubated overnight. Subsequently, SH-SY5Y cells were treated with H_2_O_2_ or CP, in the presence or in the absence of PEMFs, with three different schemes of PEMF application: (i) 24 h PEMFs continuously with H_2_O_2_ and CP; (ii) pretreated with PEMFs for 4 h, and then continuously with PEMFs, H_2_O_2_, and CP for 24 h; (iii) 24 h PEMFs continuously with H_2_O_2_ and CP and 6 h after the termination of PEMF exposure (Scheme 1). N9 cells were treated for 24 h with continuous PEMFs and CP (Scheme 1). At the end of the incubation period, MTS solution was added to each well and the optical density of each well was read on a spectrophotometer at 490 nm.

### 4.11. Nuclear Hoechst 33342 Staining

For nuclear staining with Hoechst 33342, SH-SY5Y and N9 cells were seeded in 8-well culture/chamber slides (2 × 10^5^ cells/well) and incubated overnight. Cells were treated with 1 mM of H_2_O_2_ or with 20 µM CP for 90 min with and without PEMFs. For the PEMF-exposed experimental cell group, the culture/chamber slides were positioned inside the PEMF exposure system during the H_2_O_2_ or CP treatment time. At the end of the treatments, cells were washed twice with PBS and fixed with a 4% paraformaldehyde solution for 20 min at RT. After two washes with PBS, cells were incubated with 1 µg/mL of Hoechst 33342 solution for 20 min at RT. From this step, cells were processed in the dark. Finally, cells were washed (2 times with PBS) and after removing the chamber, the slide was mounted with an antifade solution. Images were acquired with the Nikon Eclipse 5i fluorescent microscope using the UV filter. For the nuclei quantification, the images acquired were analyzed and total and altered nuclei were quantified from each image with the support of NIS Elements v 5.11 software to apply grids to the images and count the manual measurements selected options.

### 4.12. Statistical Analysis

All values included in the figures and text are expressed as mean ± standard error of the mean (SEM) of at least three independent experiments performed in duplicate. Data sets were examined by one-way analysis of variance (ANOVA) and Sidak’s multiple comparison test (*) or Student’s *t*-test (#). A *p*-value less than 0.05 was considered statistically significant.

## 5. Conclusions

This study shed light on the protective effects of PEMFs in oxidative stress occurring in AD. Collectively, the results of this work may provide a basis for additional investigation into the mechanisms behind the effects of PEMFs at different frequencies and exposure times on intracellular ROS levels in neuronal and microglial signaling networks, hence facilitating a more comprehensive analysis. In addition, future comparison of PEMF effects with other antioxidant or neuroprotective treatments would provide better context for their effectiveness. We suggest that the current research may provide insight into future studies targeting molecular, biochemical, and cellular mechanisms triggered by exposure to PEMFs.

## Data Availability

Data will be made available on reasonable request.
